# Emotional regulation and self-perceived quality of life in high-performance mountain sports athletes

**DOI:** 10.3389/fpsyg.2024.1370124

**Published:** 2024-04-16

**Authors:** Pablo Rojo-Ramos, Carmen Galán-Arroyo, Santiago Gómez-Paniagua, Antonio Castillo-Paredes, Jorge Rojo-Ramos

**Affiliations:** ^1^Physical Activity for Education, Performance and Health (PAEPH) Research Group, Faculty of Sports Sciences, University of Extremadura, Cáceres, Spain; ^2^Physical and Health Literacy and Health-Related Quality of Life (PHYQoL), Faculty of Sport Science, University of Extremadura, Cáceres, Spain; ^3^BioẼrgon Research Group, University of Extremadura, Cáceres, Spain; ^4^Grupo AFySE, Escuela de Pedagogía en Educación Física, Facultad de Educación, Investigación en Actividad Física y Salud Escolar, Universidad de Las Américas, Santiago, Chile

**Keywords:** emotional regulation, quality of life, high-level athletes, natural environment, physical activity

## Abstract

Emotional regulation is an indispensable capacity for human beings, so that alterations in it can lead to the appearance of psychological, social and/or cognitive disorders. Therefore, possessing adequate emotional strategies is intimately related to the quality of life that a person presents. In this sense, high-level athletes suffer constant setbacks and frustrations due to the performance of their sporting activity, in addition to continuous modifications of their daily life activities. Thus, the objective of this research is to explore the emotional regulation and self-perceived quality of life of high-level athletes in mountain sports, analyzing the possible influences of gender, demographic location, body mass index and age. Fifty-four athletes belonging to the High Performance Technification Center of Cáceres (Extremadura, Spain) completed a sociodemographic questionnaire, as well as the Cognitive Emotion Regulation Questionnaire and the WHOQOL-BREF. The Shapiro–Wilkins test was used to analyze the normality of the variables collected and nonparametric statistics were used since the assumption was not met. Both gender and demographic location showed significant differences in the dimensions of the two questionnaires. Likewise, age was associated with the dimensions of both scales, but not body mass index, which was only associated with self-perceived quality of life. In addition, the stepwise linear regression model predicted self-perceived quality of life with a value of 60% across self-culpability, gender body mass index and planning. Therefore, it appears that gender, demographic location, age and body mass index could exert modifications on the levels of emotional regulation and self-perceived quality of life of high-level mountain athletes.

## 1 Introduction

Emotions are a crucial part of the human condition. Without them, there would be no such thing as the exhilaration of victory or the agony of defeat (Ford and Gross, 2018). Learning to regulate them is crucial in the pursuit of well-being, in fact Koechlin et al. (2018), conclude that people who rate themselves as effective in regulating their emotions also report less negative affect and better quality of life. Valenzuela and La Portillo (2018) in their study, conclude that a relationship can be observed between people’s emotional intelligence and the achievement of the goals they set for themselves, just as Vaquero Solis et al. (2018), who deduce from the results obtained in their study that physical activity (PA), motivation levels and adaptability or emotional regulation are closely related. Psychologists Salovey and Mayer (1990) coined the concept of “emotional intelligence,” being directly linked to the term emotional regulation which constitutes the key process of emotional intelligence. This refers to those processes by which people exert an influence on the emotions they have, when they have them and how they experience and express them (Gross, 1999). Likewise, the regular practice of PA has a direct impact on emotional regulation and quality of life (Barbosa Granados and Urrea Cuéllar, 2018). Since the beginning, human beings have lived through movement, whether for utilitarian or recreational purposes. In contrast, nowadays sedentary lifestyles have become part of people’s lives, bringing with them a decrease in health, not only physical but also psychological (García Matamoros, 2019). The World Health Organization (WHO) (OMS, 2022) defines PA as any bodily movement produced by skeletal muscles, with consequent energy consumption. It further adds that PA refers to any movement, including during leisure time, to move to and from certain places, or as part of a person’s work.

Both PA and emotional regulation are an essential part of a person’s quality of life, so it is important to reject behaviors that diminish it (Ahrendt et al., 2016) and to stay active (Perea-Caballero et al., 2020). The widespread use of the term “quality of life” and research on it developed since the 1970s (Ramírez-Coronel et al., 2021). Years later, the WHO (OMS, 1998) defined it as individuals’ perceptions of their position in life in the context of the culture and value systems in which they live and in relation to their goals. Additionally, the transition to adolescence is marked by enormous changes in social, biological and personality development (Brandes et al., 2020), with some of the main challenges of this stage being the lack of social discipline or the influence of negative emotions from family members (Verdecia et al., 2018). Thus, the increase in the quality of life in adolescence lies in leaving behind sedentary lifestyles, adopting healthy and higher quality dietary patterns or behaviors, as evidence has been found of their importance in increasing it (Wu et al., 2019). The latest research, the importance of adolescence as a critical period for the development of emotional regulation has been recognized by several investigations (Lennarz et al., 2019), as for example shown by Silva et al. (2018), where they specify that the level of affect, expressive suppression and cognitive reappraisal influence the emotional regulation of adolescents and their daily lives. Adaptive processes and changes that occur within adolescence (Verdecia et al., 2018), as well as interpersonal relationships, are complex processes that require self-analysis, however, that capacity is not completely internalized (Bernal et al., 2018), hence the importance of emotional regulation at this stage of life.

As discussed, being physically active is one of the most important things people can do to improve physical and mental health (OECD, 2023). However, sedentary lifestyles and the rise of social networks, as well as the way adolescents communicate and interact nowadays, have contributed to a decrease in the level of PA, which is associated with a greater risk of suffering alterations in the psychological, emotional and social state of adolescents (Mascia et al., 2020). In summary, the practice of PA prevents the onset of diseases (Warburton and Bredin, 2016), which consequently leads to an increase in the quality of life (Yagüe Nogué et al., 2021), which in turn allows adolescents to acquire higher levels of emotional regulation (Simón-Saiz et al., 2018). In this sense, during the last few years and in favor of achieving a high quality of life, there has been growing interest in the positive benefits that can be obtained from natural environments and time spent outdoors (Ballester Martínez et al., 2022). PA carried out in nature brings with it a positive impact and even has synergistic effects (Eigenschenk et al., 2019). In fact, in the systematic review by Ballester Martínez et al. (2022) on studies related to PA, nature and mental well-being, the existence of a significant influence of PA in nature on the psychological well-being of the participants is shown.

In this context, there are numerous studies that analyze the relationships between emotional regulation or quality of life (Hervás and Moral, 2017; Lennarz et al., 2019; Salinas Ponce and Villacres, 2021), as well as the influence that PA has on these psychological issues, however these terms have been little explored in high performance athletes who develop their activity in the natural environment. Therefore, the aim of this study is to analyze emotional regulation and quality of life, as well as their relationship with sex, age and body mass index (BMI), in athletes of mountain sports modalities who carry out their activity regularly in the natural environment. Consequently, the main hypothesis for this study would be: There is a significant direct relationship between emotional regulation and quality of life in high-performance mountain athletes and the secondary hypothesis would be: Variables of sex, demographic location, age and body mass index could exert modifications on the levels of emotional regulation and self-perceived quality of life of high-level mountain athletes.

## 2 Materials and methods

### 2.1 Participants

The sample was selected using the non-probabilistic sampling method based on convenience sampling (Salkind et al., 1999). Of the total sample (*n* = 54), 68.5% were boys and 31.5% were girls, so it can be considered a gender-balanced sample. The inclusion criteria of the participants were to have the accreditation of technification, high performance or high-level athletes in any of the disciplines considered as mountain sports by the Spanish Federation of Mountaineering and Climbing.

To characterize the sample (Table 1), other variables were defined, such as level of education, demographic location, considering rural centers to be those with less than 20,000 inhabitants, and the sports modality of the athletes. The mean age was 21.78 years (SD = 7.88) and the mean BMI was 20.99 (SD = 2.64), calculated from the height and weight data self-reported by the participants.

**Table 1:** Sample characterization (*N* = 54).

Variable	Categories	*N*	%
Sex	Men	37	68.5
	Women	17	31.5
Education level	Secondary education	28	51.9
	Professional training	8	14.8
	University	10	18.5
	Master’s or doctorate	8	14.8
Demographic location	Rural environment	23	42.6
	Urban environment	31	57.4
Athlete’s condition	Technification	23	42.6
	High performance	28	51.9
	High-level	3	5.6

N, number; %, percentage; SD, standard deviation; M, Mean.

### 2.2 Procedure

The method of data collection was by digital means and the technique used was the realization of self-administered questionnaires, this type of technique facilitates the collection of data to be able to work with them later, saving time and costs (Anderson and Kanuka, 2003). Its main advantage is the possibility of being able to carry them out remotely, in this case the questionnaire was elaborated with the digital application Google Forms.

The questionnaire was completely anonymous and consisted of three parts, two of which were the instruments and one of which was the instruction sheet for proper understanding. The average completion time was about 3 min. All data were collected between October 2022 and March 2023.

It was distributed through telephone contacts, social networks and by email to the various federations that have athletes at the National Technification Center in Cáceres (Spain).

### 2.3 Instruments

First, a questionnaire was designed with six sociodemographic questions (sex, age, demographic location, height, weight and sport modality) through self-reporting. The BMI (kg/m^2^) was obtained by applying the following formula: BMI = weight in kilograms / (height in meters)^2^.

Emotional regulation was also assessed using the Cognitive Emotion Regulation Questionnaire (CERQ) (Garnefski et al., 2001). The CERQ instrument is composed of 36 items that evaluate nine cognitive-emotional coping strategies for dealing with stressful situations and events. This scale is based on a 5-point Likert-type scale, where 1 is “Sometimes” and 5 is “Always.” The dimensions that make up the questionnaire are the following: (1) Self-blame (e.g., “I feel that I am to blame for what happened”); (2) Acceptance (e.g., “I think I have to accept what has happened”); (3) Rumination (e.g., “I often think about how I feel in relation to what has happened to me”); (4) Positive focus (e.g., “I think of something more pleasant than what has happened to me”); (5) Planning (e.g., “I think about what is the best thing I could do”); (6) Positive reassessment (e.g., “I think I can learn something from the situation”); (7) Perspective taking (e.g., “I think it could have been much worse”); (8) Catastrophism (e.g., “I often think that what has happened to me is much worse than what has happened to other people”); and (9) Blame others (e.g., “It seems to me that others are to blame for what happened”). Similarly, these 9 dimensions can be grouped into adaptive strategies (Acceptance, Positive focus, Planning, Positive reassessment and Perspective taking) and disadaptive strategies (Self-blame, Rumination, Catastrophism and Blame others). The internal consistency reported through Cronbrach’s alpha in the different subscales ranges from 0.68 (Blame others) to 0.83 (Rumination). Also, in the Spanish version for adolescents (Chamizo-Nieto et al., 2020), the internal consistency of the different subscales ranges from 0.62 (Catastrophism) to 0.83 (Positive focus).

Finally, the self-perceived quality of life was analyzed using the WHOQOL-BREF (Nejat et al., 2006). The instrument has 26 items consisting of four domains: (1) Physical health (7 items), including items on mobility, daily activities, functional ability, energy, pain, and sleep; (2) Psychological health (6 items), referring to self-image, negative thoughts, positive attitudes, self-esteem, mentality, learning ability, memory concentration, religion and state of mind; (3) Social relationships (3 items), complementing information on personal relationships, social support and sex life; and (4) Environmental health (8 items); covering issues related to financial resources, safety, health and social services, physical living environment, opportunities for acquiring new skills and knowledge, recreation, general environment (noise, air pollution, etc.) and transportation. Each individual item of the questionnaire is scored from 1 to 5 on a Likert-type response scale, and the scores for each dimension are then transformed into a scale from 1 to 100.

### 2.4 Statistical analysis

Prior to the analysis, 3 negative WHOQOL-BREF items were inverted to unify the analysis domain. Then, to determine the type of statistical tests to be used, the distribution of the data was explored to see if the normality assumption was met using the Shapiro–Wilk test, since a sample size of around 50 participants was obtained (Mendes and Pala, 2003). This test determined that this assumption was not met, so it was decided to use nonparametric statistical tests.

In order to analyze the differences between the scores of each of the dimensions according to sex or demographic location, the Mann Whitney U test was used, establishing a significance level of *p* < 0.05. Also, to determine the degree of relationship between each of the dimensions and age or BMI, Spearman’s Rho test was used. For the interpretation of this statistic, the ranges established by Mondragón Barrera (2014) were taken into account: coefficients between 0.01 and 0.10 determine the existence of a low correlation, values between 0.11 and 0.50 imply a medium degree of correlation, from 0.51 to 0.75 a strong correlation, from 0.76 to 0.90 a high correlation, and above 0.91 the correlation is perfect.

In addition, a simple stepwise regression test was used to analyze changes in quality of life taken as a construct consisting of the dimensions that make up the WHOQOL-BREF in relation to the CERQ dimensions, gender and BMI of the participants. A significance level of less than 0.05 was required to enter the variables in the predictive model.

Finally, Cronbach’s Alpha was used to analyze the reliability of the instrument. According to Nunnally and Bernstein (1994), reliability values between 0.60 and 0.70 can be considered acceptable, while values between 0.70 and 0.90 can be considered satisfactory.

## 3 Results

Table 2 shows the descriptive data for each of the CERQ dimensions according to sex and demographic location based on the mean and standard deviation. The statistical significance was obtained from the Mann–Whitney U test to analyze differences between groups.

**Table 2:** Descriptive analysis and differences in the CERQ dimensions.

Dimension	Sex			Demographic location		
	Men	Women		Rural	Urban	*p*
	M (SD)	M (SD)	*p*	M (SD)	M (SD)	
Self-blame	3.09 (0.80)	3.38 (0.76)	0.21	3.09 (0.77)	3.25 (0.81)	0.44
Acceptance	11.02 (2.18)	11.85 (2.72)	0.26	12.26 (1.84)	10.56 (2.49)	0.01*
Rumination	10.30 (2.55)	12.10 (2.61)	0.03*	11.09 (2.41)	10.70 (2.89)	0.74
Positive focus	8.48 (2.42)	8.27 (2.89)	0.70	8.63 (2.73)	8.26 (2.45)	0.67
Planning	11.77 (2.37)	13.26 (2.68)	0.03*	13.67 (1.97)	11.17 (2.42)	0.01*
Positive reassessment	12.15 (2.90)	13.05 (3.26)	0.26	13.91 (2.93)	11.34 (2.63)	0.01*
Perspective taking	10.97 (2.17)	11.45 (2.93)	0.41	11.48 (2.76)	10.86 (2.14)	0.43
Catastrophism	7.80 (2.73)	7.17 (2.96)	0.29	7.56 (3.13)	7.83 (2.57)	0.68
Blame others	7.35 (2.42)	6.50 (2.19)	0.30	7.13 (2.31)	7.08 (2.43)	0.81
Adaptive strategies	10.88 (1.62)	11.58 (1.94)	0.16	11.99 (1.47)	10.44 (1.64)	0.01*
Disadaptive strategies	7.13 (1.65)	7.30 (1.50)	0.68	7.22 (1.44)	7.16 (1.72)	0.94

M, Mean; SD, standard deviation. Each score obtained is based on a Likert scale (1–5). *p is significant < 0.05.

Girls scored higher than boys on the dimensions self-blame, acceptance, rumination, planning, positive reassessment, perspective taking, adaptive strategies and disadaptive strategies. In turn, boys scored higher on the dimensions positive focus, catastrophism and blame others. However, significant differences with respect to gender were only obtained in the third (Rumination) and fifth dimension (Planning). On the other hand, demographic location exhibited the highest scores in rural settings on most factors, except for self-blame and catastrophism. Statistically significant differences were also found in acceptance, planning, positive reassessment and adaptive strategies, all of which were in favor of rural settings.

Table 3 shows the scores and differences obtained in each of the dimensions of the WHOQOL-BREF instrument. With regard to sex, significant differences were observed in the psychological health dimension, with men showing higher scores in all dimensions of the scale. With regard to demographic location, significant differences were again observed in the psychological health dimension, and physical health was added. Similarly, the scores follow a clear trend, with the rural locations scoring higher except in the environment dimension.

**Table 3:** Descriptive data and differences in each dimension of the WHOQOL-BREF as a function of gender and demographic location.

Dimension	Sex			Demographic location		
	Men	Women		Rural	Urban	
	M (SD)	M (SD)	*p*	M (SD)	M (SD)	*p*
Physical Health	4.15 (0.52)	3.93 (0.58)	0.13	4.31 (0.42)	3.90 (0.56)	0.01*
Psychological Health	4.09 (0.64)	3.56 (0.65)	<0.01*	4.19 (0.53)	3.73 (0.73)	0.02*
Social realtionships	3.71 (0.75)	3.45 (0.97)	0.33	3.82 (0.79)	3.48 (0.83)	0.14
Environmental health	3.95 (0.05)	3.63 (0.65)	0.15	3.76 (0.58)	3.92 (0.57)	0.30

M, Mean; SD, standard deviation. Each score obtained is based on a Likert scale (1–5). *p is significant < 0.05.

Table 4 used Spearman’s Rho test to analyze the relationship between each of the dimensions with age and BMI. As for CERQ, age appears to be directly, mean and significantly related to positive focus and adaptive strategies. Also, with the same characteristics but inversely, the dimension of catastrophism is related to age. Likewise, BMI does not seem to be related to CERQ factors. With regard to the WHOQOL-BREF, only environmental health showed significance when associated with age and BMI, with both relationships being mean and inverse.

**Table 4:** Relationships between the dimensions of the questionnaires with age and BMI variables.

Instrument	Age *p (p)*	BMI *p (p)*
CERQ		
1. Self-blame	−0.08 (0.55)	−0.12 (0.38)
2. Acceptance	0.20 (0.14)	−0.07 (0.61)
3. Rumination	0.02 (0.83)	0.06 (0.66)
4. Positive focus	0.35 (0.01)*	0.03 (0.80)
5. Planning	0.14 (0.28)	−0.10 (0.44)
6. Positive reassessment	0.25 (0.06)	−0.04 (0.73)
7. Perspective taking	0.05 (0.72)	−0.06 (0.63)
8. Catastrophism	−0.29 (0.03)*	0.07 (0.59)
9. Blame others	−0.10 (0.46)	0.20 (0.13)
10. Adaptive strategies	0.33 (0.01)*	0.01 (0.99)
11. Disadaptive strategies	−0.17 (0.21)	0.11 (0.39)
WHOQOL-BREF		
12. Physical health	0.02 (0.88)	−0.13 (0.33)
13. Psychological health	0.01 (0.97)	−0.05 (0.67)
14. Social relationships	−0.07 (0.61)	−0.02 (0.88)
15. Environmental health	−0.33 (0.01)*	−0.43 (0.01)*

BMI, Body Mass Index. Each score obtained is based on a Likert scale (1–5). *p is significant < 0.05.

Table 5 shows a model for predicting quality of life using the simple regression test. This predictive model (perceived quality of life scores = 0.057 x Planning – 0.312 x Self-blame – 0.052 x BMI – 0.374 x Gender) shows a predictive capacity for changes in quality of life of approximately 60%, with R2 being 0.60.

**Table 5:** Model predicting changes in quality of life.

	Model 1 (R2) = 0.60			
Variable	*β*	SE	*t*	*p*
Self-blame	−0.312	0.079	−3.923	<0.01
BMI	−0.052	0.024	−2.175	0.03
Gender	−0.374	0.137	−2.742	0.01
Planning	0.057	0.025	2.270	0.03
Constant	5.358	0.676	7.927	<0.01

Finally, Table 6 shows the Cronbach’s alpha values reported for each of the CERQ and WHOQOL-BREF dimensions. Even with a small sample, satisfactory values were obtained (between 0.70 and 0.90), except in the social relations dimension.

**Table 6:** Cronbach’s alpha coefficients for each dimension of the scales.

Instrument	Cronbach’s alpha
CERQ	
1. Self-blame	0.70
2. Acceptance	0.72
3. Rumination	0.77
4. Positive focus	0.72
5. Planning	0.72
6. Positive reassessment	0.85
7. Perspective taking	0.71
8. Catastrophism	0.70
9. Blame others	0.74
10. Adaptive strategies	0.70
11. Disadaptive strategies	0.70
WHOQOL-BREF	
12. Physical health	0.70
13. Psychological health	0.74
14. Social relationships	0.60
15. Environmental health	0.70

## 4 Discussion

The purpose of this study was to determine the influence of emotional regulation on the quality of life of high-performance athletes. In the analysis of the main hypothesis between emotional regulation and self-perceived quality of life in athletes, no significant relationships were found between the dimensions; this may be due to the low sample size and low internal consistency of the items and dimensions of the instruments used. However, differences were found between different items and dimensions of the instruments used.

Emotional regulation as an influential factor in the quality of life of athletes is an aspect that has been addressed in several studies, inferring that, possessing emotional regulation strategies helps to improve mental well-being in the daily lives of people (Lennarz et al., 2019; Bird et al., 2021) and in this case athletes (Tamminen et al., 2021). Furthermore, in the study by Ono et al. (2019), testimonies of athletes are shared in which they consider that the pressure of training combined with the rest of daily tasks generates a mental impact on their life. Likewise Simón-Saiz et al. (2018) also show that resilience, an aspect closely linked to emotional regulation, generates a positive effect on quality of life, being able to conclude then that the acquisition of emotional regulation strategies helps athletes to improve it. Regarding the gender variable, the scientific literature provides information in which, as in this study, there are differences between the male and female sexes in emotional regulation processes (Kwon et al., 2013; Dixon-Gordon et al., 2015). Goubet and Chrysikou (2019) demonstrated in their research results that women possessed a significantly higher repertoire than men, suggesting that they may have access to a greater number of strategies, coinciding with the results obtained in the present study where girls presented higher scores in the different dimensions of the CERQ. On the other hand, the influence of demographic location on emotional regulation has not been a focus extensively investigated by the authors. On this issue there are contrary results, on the one hand some research shows that the place of residence influences emotional regulation (Kar et al., 2014) while on the other hand there are studies that reflect that there is no significant evidence between both aspects (Kant, 2019; Sørensen, 2021).

Since the WHO created the WHOQOL-BREF instrument, several authors have made use of this tool to determine the quality of life of people in a given context. In this study, the relationships that exist between the sex and demographic location of the participants and their quality of life have been verified, revealing that there are differences between both sexes in relation to the “psychological health” dimension. Several studies corroborate this result (Fisk, 2018; Esteban-Gonzalo et al., 2020; Walton et al., 2021) concluding that gender may be a factor influencing quality of life. On the other hand, in the present study higher levels of quality of life in most dimensions are seen in the rural setting (Cai and Wang, 2018) also obtained similar results, while in the study of (Sompolska-Rzechula and Kurdys-Kujawska, 2020) the results show significant variation between quality of life between rural and urban settings.

Using Spearman’s Rho test, the relationship between the different dimensions of the CERQ and the WHOQOL-BREEF was analyzed to see if age and BMI influenced them. The results shown in this study linked to age and the different dimensions of the CERQ hint that there is some relationship between them, coinciding with the results of different studies (Deng et al., 2019; Perry et al., 2019; Burr et al., 2021) in which it is shown that there is a significant relationship between these two aspects, with a large number of age-related changes occurring in emotional regulation during adolescence (Deng et al., 2019). In contrast, the values found in the CERQ results focused on BMI show that no relationship is found with the dimensions of that instrument. No research has been found that specifically addresses the influence of emotional regulation on BMI in high-performance athletes, but evidence of some influence of emotional regulation on BMI can be found in people who practice PA at a non-professional level (Jones et al., 2019; Ruzanska and Warschburger, 2019). Quality of life measured through the WHOQOL-BREF instrument has also been the subject of study with a focus on how age and BMI affect it. In our study we only found a relationship with both variables in the environmental health dimension, as Wallas et al. (2019), whose results provide an environmental influence on BMI, or Lange et al. (2011) who state that environmental factors are significantly associated with adolescent BMI.

Quality of life is a concept that is determined by multiple factors involving physical, psychological and social aspects (Irigaray and Trentini, 2009). In this research, a prediction of change in quality of life of 60% was found based on the correlation between variables such as self-culpability, BMI, sex and planning. Although there are not many findings in the scientific literature on the correlation between these four elements in favor of quality of life, we do find studies such as that of Kyeong et al. (2020) in which it is explained that those with low quality of life may be more vulnerable to being negatively affected by self-criticism. On the other hand, different research has found findings of a correlation between sex and BMI in different populations (Aksoydan and Çakır, 2011; Nwizu et al., 2011).

Therefore, the first hypothesis was declined but the second hypothesis has been reaffirmed.

### 4.1 Limitations

Some limitations can be found in this study. The research was carried out by means of non-probabilistic convenience sampling, which did not fully ensure the representativeness of the sample and could have generated various biases. Likewise, another limitation of the study is the low sample size, which could have limited the results. Likewise, data collection was done through online surveys being faster and cheaper than standard surveys but may entail disadvantages such as low response rates (Iversen et al., 2020).

### 4.2 Future lines of research

Future research could try to extend the information in the current scientific literature on the correlation between dimensions such as self-culpability, planning and aspects such as gender and BMI and how they influence the quality of life of a larger sample of high-performance athletes. Similarly, a line of research could be opened in relation to the rural or urban environment and how it influences the quality of life of athletes.

## 5 Conclusion

The influence of emotional regulation on the quality of life of high-performance athletes was the main object of analysis of this study and results were found that may indicate that emotional regulation influences the quality of life of these athletes, although not significantly. Women scored higher on some of the CERQ dimensions, indicating that they may possess more regulation strategies than men. In addition, demographic location, age and body mass index could exert modifications on the levels of emotional regulation and self-perceived quality of life of high-performance athletes. Studies such as this one allow a better understanding of the importance of the application of emotional regulation strategies and it would be interesting for coaches to be aware of them in order to favor not only the performance of athletes, but also to increase their mental well-being and their self-perceived quality of life.

## Data availability statement

The raw data supporting the conclusions of this article will be made available by the authors, without undue reservation.

## Ethics statement

The use of these data did not require approval from an accredited ethics committee, as they are not covered by data protection principles, i.e., they are non-identifiable, anonymous data collected through an anonymous survey for teachers. In addition, based on Regulation (EU) 2016/679 of the European Parliament and of the Council on 27 April 2016 on the protection of individuals concerning the processing of personal data and on the free movement of such data (which entered into force on 25 May 2016 and has been compulsory since 25 May 2018), data protection principles do not need to be applied to anonymous information (i.e., information related to an identifiable natural person, nor to data of a subject that is not, or is no longer, identifiable). Consequently, the Regulation does not affect the processing of our information. Even for statistical or research purposes, its use does not require the approval of an accredited ethics committee. The informed consent of the subjects participating in the study was not necessary, as the data were collected anonymously and there were no minors under 14 years of age. Therefore, and in compliance with paper 13.1 of the LOPD Regulation, which states that “in the case of minors under fourteen years of age, the consent of the parents or guardians shall be required”, although the document was created, it was not required.

## Author contributions

PR-R: Conceptualization, Investigation, Project administration, Resources, Writing – original draft, Writing – review & editing. CG-A: Investigation, Resources, Supervision, Validation, Visualization, Writing – original draft, Writing – review & editing. SG-P: Investigation, Resources, Writing – original draft, Writing – review & editing, Methodology, Software. AC-P: Supervision, Visualization, Writing – original draft, Writing – review & editing, Funding acquisition, Investigation, Resources. JR-R: Methodology, Software, Writing – original draft, Writing – review & editing, Data curation, Project administration, Supervision, Visualization.
